# *De novo* Transcriptome Analysis of *Rhizoctonia solani* AG1 IA Strain Early Invasion in *Zoysia japonica* Root

**DOI:** 10.3389/fmicb.2016.00708

**Published:** 2016-05-18

**Authors:** Chen Zhu, Lin Ai, Li Wang, Pingping Yin, Chenglan Liu, Shanshan Li, Huiming Zeng

**Affiliations:** ^1^Biochemistry and Molecular Biology Department, College of Biological Sciences and Technology, Beijing Forestry UniversityBeijing, China; ^2^Ecology Department, College of Forestry, Beijing Forestry UniversityBeijing, China; ^3^Silviculture Forestry Department, College of Forestry, Beijing Forestry UniversityBeijing, China; ^4^Turfgrass Management Department, College of Forestry, Beijing Forestry UniversityBeijing, China

**Keywords:** *Zoysia japonica* steud, *Rhizoctonia solani* AG1 IA strain, brown spot, RNA sequencing, transcriptome analysis

## Abstract

*Zoysia japonica* brown spot was caused by necrotrophic fungus *Rhizoctonia solani* invasion, which led to severe financial loss in city lawn and golf ground maintenance. However, little was known about the molecular mechanism of *R. solani* pathogenicity in *Z. japonica*. In this study we examined early stage interaction between *R. solani* AG1 IA strain and *Z. japonica* cultivar “Zenith” root by cell ultra-structure analysis, pathogenesis-related proteins assay and transcriptome analysis to explore molecular clues for AG1 IA strain pathogenicity in *Z. japonica*. No obvious cell structure damage was found in infected roots and most pathogenesis-related protein activities showedg a downward trend especially in 36 h post inoculation, which exhibits AG1 IA strain stealthy invasion characteristic. According to Gene Ontology (GO) and Kyoto Encyclopedia of Genes and Genomes (KEGG) database classification, most DEGs in infected “Zenith” roots dynamically changed especially in three aspects, signal transduction, gene translation, and protein synthesis. Total 3422 unigenes of “Zenith” root were predicted into 14 kinds of resistance (R) gene class. Potential fungal resistance related unigenes of “Zenith” root were involved in ligin biosynthesis, phytoalexin synthesis, oxidative burst, wax biosynthesis, while two down-regulated unigenes encoding leucine-rich repeat receptor protein kinase and subtilisin-like protease might be important for host-derived signal perception to AG1 IA strain invasion. According to Pathogen Host Interaction (PHI) database annotation, 1508 unigenes of AG1 IA strain were predicted and classified into 37 known pathogen species, in addition, unigenes encoding virulence, signaling, host stress tolerance, and potential effector were also predicted. This research uncovered transcriptional profiling during the early phase interaction between *R. solani* AG1 IA strain and *Z. japonica*, and will greatly help identify key pathogenicity of AG1 IA strain.

## Introduction

*Rhizoctonia solani* (teleomorph: *Thanatephorus cucumeris*) is one kind of soilborne basidiomycete fungus, which causes diseases like sheath blight, aerial blight, and brown spot in many monocots and dicots plants (Hane et al., 2014). Brown spot in *Zoysia japonica* is a destructive fungal disease caused by AG1 IA strain, one main member of AG1 subgroup in *R. solani* 14 anastomosis group (AG1 to AG13 and AGB1) (Foley et al., 2013), which causes annual substantial finance loss in city lawn and golf ground maintenance.

The necrotrophic lifestyle of AG1 IA strain confers itself with strong adaptiveness in various environmental conditions and robust invading ability in multiple kinds of plants (Venu et al., 2007). To date, rice sheath blight is a well-studied case which unveiled AG1 IA strain invasion process in morphological and anatomical aspects (González-Vera et al., 2010). There are four major stages for its early invasion in rice including adhesion, penetration, colonization, and host reaction, which is similar in *Z. japonica* infection according to our recent observation. Major breaking through in discovering the key part in pathogenicity is the identification in its effector proteins which were delivered into host plant to establish parasitic relationship (Zheng et al., 2013). In return, these effector proteins sometimes triggered host recognition and resulted in effector-triggering immunity in host plant. Many researches were done during past decades to detect the effector proteins during AG1 IA strain invasion, however there is still little known in this part. Recently, with genome sequencing of AG1 IA strain, large group of genes encoding secreted proteins, enzymes in secondary metabolism, carbohydrate-active enzymes and transporters were annotated which indicates its necrotrophic lifestyle. Many genes of AG1 IA strain were also predicted as potential plant “effector” and virulence associated factor in rice sheath blight symptome. Three kinds of novel secreted effectors, glycosyltransferase GT family 2 domain, cytochrome C oxidase assembly protein CtaG/cox11 domain and peptidase inhibitor I9 domain were verified (Zheng et al., 2013). Thus, future work in discovering the molecular details in AG1 IA strain pathogenicity becomes much more possible with genome information.

Molecular breeding for anti-AG1 IA strain cultivar is also another hit zone during past decades. Pathogenesis-related proteins such as chitinase, NADPH oxidase, thaumatin-like protein and β-1,3-glucanase encoded genes under control of cauliflower mosaic virus 35 s in rice or *Arabidopsis* were over-expressed, which confers improved resistance against *R. solani* invasion (Molla et al., 2013). However, these proteins were not host-derived signal perception factors, which also caused metabolic disturbance in plant. Therefore, finding effector proteins of *R. solani* strain and host sensor proteins (host-derived signal perception) is the efficient way for future anti-*R. solani* breeding work.

Until recently, no effective measures can be done to control brown spot in *Z. japonica* except using universal fungicide, which negatively caused evolution fungicide resistance. Thus, *Z. japonica* anti-*R. solani* AG1 IA strain cultivar is badly needed, which can be applied by understanding molecular mechanism of AG1 IA pathogenicity in *Z. japonica* and finding key candidate sensor proteins for *Z. japonica*.

In this paper, we checked *R. solani* AG1 IA strain during 12–48 h infection and 12–48 h post *R. solani* AG1 IA strain inoculated (hp*Rs*-i) *Z. japonica* cultivar “Zenith” root (*R. solani* AG1 IA strain un-inoculated (*Rs*-ui) “Zenith” root as control) by cell ultra-structure analysis, pathogenesis-related protein assay and transcriptome analysis to seek clues for AG1 IA strain pathogenicity in “Zenith.”

## Materials and methods

### Biological material, infection procedures, and time-course infection

The *R. solani* AG1 IA strain was cultured on potato dextrose broth medium at 25°C for 7 days in the dark. Sterilized seedlings of *Z. japonica* cultivar “Zenith” were grown on MS plates for 7 weeks. Root inoculated with AG1 IA strain was carried out as previously described by Rfael Perl-Treves (Perl-Treves et al., 2004). The moist millets co-cultured with AG1 IA strain for 7 days at 25°C in the dark were placed directly beside the roots. Roots were harvested at 12, 24, 36, 48 h post-inoculation and un-inoculated “Zenith” roots were treated as control.

### RNA extraction, library construction, and RNA-sequencing

Twelve to forty-eight hp*Rs*-i “Zenith” roots RNA was extracted using TRIzol reagent according to the manufacturer's protocol (Invitrogen, USA). The isolated RNA samples were sent to Gene Denovo Co. (Guangzhou, China) for libraries construction, RNA sequencing, and unigene annotation. Libraries were established using the Illumina kit. The cDNA library was sequenced on the Illumina HiSeq™ 2500 sequencing platform.

### *De novo* transcriptome assembly and annotation

The datasets were processed by removing adaptor sequences, empty reads and low-quality sequences with threshold values of Q30. Then clean reads of each sample were assembled using Trinity platform (http://trinityrnaseq.sourseforge.net/) to obtain corresponding transcripts (Grabherr et al., 2011). The uni-transcripts were then clustered by using TGICL assembly strategy (Pertea et al., 2003). AG1 IA strain uni-transcripts were isolated by using alignment to genome short gun data Rhisol_AG1IA (DDBJ/EMBL/GenBank under the accession code AFRT00000000). Each uni-transcript was normalized into RPKM (reads per kilobase of exon model per million mapped reads) values (Mortazavi et al., 2008). Uni-transcript abundance was calculated by the ratio of RPKM values. The false discovery rate (FDR) control method was applied to identify the threshold of the *P*-value in different uni-transcripts abundance (Reiner et al., 2003). Uni-transcripts with FDR < 0.01 and Fold change ≥ 2 were treated as differential expression unigenes (DEGs) and unigenes of two species were further annotated by NCBI non-redundant (Nr), the Swiss-prot, Gene Ontology (GO) and Kyoto Encyclopedia of Genes and Genomes (KEGG), Plant Resistance Gene Database (PRGdb), Pathogen Host Interaction (PHI), Pfam database.

### Validation of DEGs expression profile

Total RNA in each three individual biological replicate samples of T10, T11, T12, T13, T14 were transcripted by using SuperScript II reverse transcriptase (Invitrogen, USA). The cDNA solution was then 10-fold diluted into working solution before real time quantitive PCR (RT-qPCR) assay. Primers of randomly selected six unigenes of “Zenith” roots (*methionine aminopeptidase 2A, ribosomal RNA processing, abscisic acid 8*′*-hydroxylase, peroxidase 5, light-inducible protein CPRF2, cytochrome c oxidase subunit 1*) were designed by PrimerPremier5 software and RT-qPCR was performed as previously described (Hacquard et al., 2010). The RT-qPCR data was collected by StepOnePlus software. Unigenes expression was normalized against the expression levels of the *Actin* gene with 2^−Δ*ΔCT*^ method. Primer sequences are presented in Supplementary Table 3.

### Transmission electron microscope analysis

Twelve to forty-eight hp*Rs*-I “Zenith” roots and *Rs*-ui “Zenith” root fresh segments (not larger than 1 mm^3^) were collected and fixed for 12 h with 2.5% (v/v) glutaraldehyde in 0.1 M phosphate–citrate buffer (pH 7.0). Post fix samples were treated with 2% osmium tetroxide at room temperature for 1–2 h, and then washed for 2 h with phosphate–citrate buffer. The samples were dehydrated by using a grade series of ethanol and embedded in Spurr's resin. Ultrathin sections were processed as previously described (Weigel and Glazebrook, 2010). The sections were observed and photographed under transmission electron microscopy (H-7650) at an operating voltage of 80 kV.

### Pathogenesis-related protein activity assay

Pathogenesis-related proteins were checked in 12–48 hp*Rs*-I “Zenith” roots (each experiment used three biological replications, *Rs*-ui “Zenith” root was treated as control). The phenylalanine ammonia-lyase (PAL) activity was determined by extracting supernatant from 0.1 g homogenized “Zenith” root in 100 mM phosphate buffer pH 6.0, 2 mM EDTA, 4 mM dithiothreitol, and 2% (w/w) polyvinylpyrrolidone. The supernatant was used for determination of protein concentration (Bradford, 1976). The absorbance of 0.2 ml supernatant with 2 ml of 0.01 M borate buffer (pH 8.7) and 1 ml of 0.02 M L-phenylalanine (pre-dissolved in 0.01 M borate buffer pH 8.7) after 60 min at 30°C was measured at 290 nm (Kovácik et al., 2009). One unit of PAL activity was defined as the amount of PAL in 1 g protein that produced 1 nanomole of cinnamic acid in 1 s as U/g. The chitinase activity was calculated with supernatant from 0.1 g homogenized root in 3.0 mL of ice-cold sodium acetate buffer (pH 5.0). 1.0 mL supernatant with 2.0 mL 0.1 M sodium acetate buffer (pH 5.0) and 0.1 g colloidal chitin was incubated at 37°C for 12 h. The supernatant was also used for determination of protein concentration (Bradford, 1976). The reaction was terminated by boiling water bath for 10 min then waiting for its cooling down to room temperature. Then centrifuge it at 1500 g for another 5 min. The 2.0 mL supernatant was mixed with 2.0 mL dinitrosalicylic acid reagent, boiling for 10 min. Then 6.0 mL distilled water was added, and absorbance read at 540 nm was measured (Su et al., 2014). One unit of chitinase activity was defined as the amount of chitinase in 1 g protein that liberates 1 nanomole N-acetamino-glucose in 1 s as U/g. The β-1,3-glucanase activity was measured by monitoring standard assay mixture containing 0.25% (w/v) Laminaria digitata laminarin, 50 mM sodium acetate, pH 5.5, and enzyme in a total volume of 50 μL was incubated at 37°C (Ballhorn et al., 2014). β-1,3-glucanase activity was defined as certain amount of β-1,3-glucanase in 1 g protein required to release 1 nanomole glucose equivalents in 1 s as U/g. The lipoxygenase (LOX) activity was determined by using 0.1 g plant root homogenized in 0.2 M boric acid buffer at pH 7.0. The homogenate was centrifuged at 12,000 g for 30 min, while the supernatant was used for determination of protein concentration (Bradford, 1976) and LOX activity. The absorbance was measured at 234 nm in reaction mixture contained 0.2 M boric acid buffer (pH 8.0), 25 μl of plant extract, and 25 μl of linoleic acid as a substrate in a final volume of 1 ml after 4 min at 30°C (Zhao et al., 2014). LOX activity was determined as amount of LOX in 1 g protein that produced 1 nanomole of cinnamic acid in 1 s as U/g.

## Result

### Cell ultra-structural analysis of “Zenith” root in early infection stage

**Figure 1:**
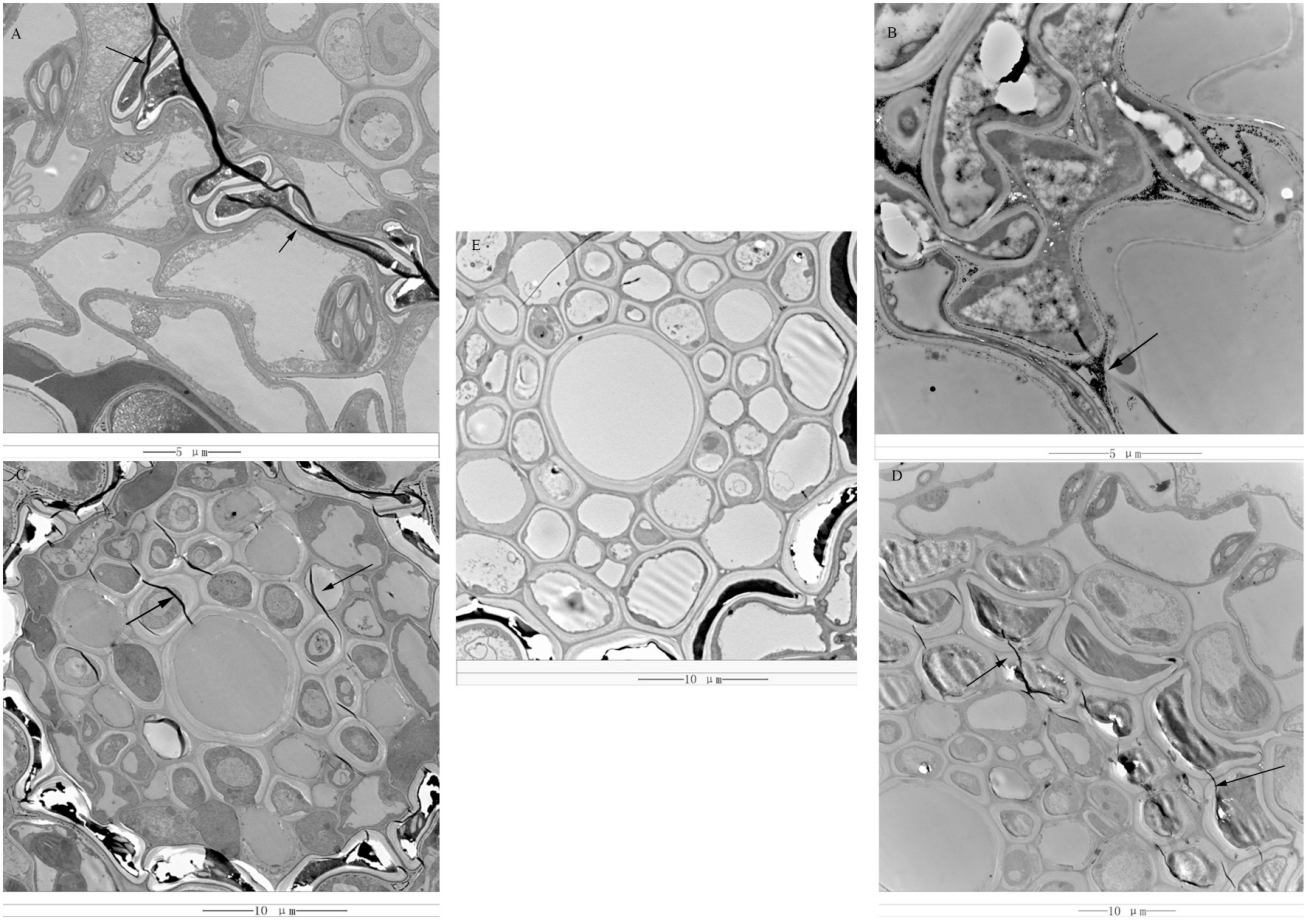
**(A)** 12 hp*Rs*-i “Zenith” root, bar = 5 μm; **(B)** 24 hp*Rs*-i “Zenith” root, bar = 5 μm; **(C)** 36 hp*Rs*-i “Zenith” root, bar = 10 μm; **(D)** 48 hp*Rs*-i “Zenith” root, bar = 10 μm; **(E)** A-ui “Zenith” root, bar = 10 μm; “Arrow,” indicated the mycelia of AG1 IA strain.

*Rs*-ui “Zenith” root and 12–48 hp*Rs*-i “Zenith” root were scanned by transmission electron microscope to discover the early invasion process of AG1 IA strain in “Zenith” (Figure 1).

*Rs*-ui “Zenith” root showed intact cell wall structure and no sign of mycelia (Figure 1E). Twelve hp*Rs*-i “Zenith” root exhibited the appearance of mycelia between cell walls (Figure 1A). The continuous invasion of AG1 IA strain mycelia penetrate the 24 hp*Rs*-i “Zenith” root cell wall and spread out into the cytoplasm (Figure 1B). The mycelia reached the center of 36 hp*Rs*-i “Zenith” root cell (Figure 1C) and surrounded the whole area of 48 hp*Rs*-i root cell (Figure 1D).

In general, the early infection of AG1 IA strain in “Zenith” root showed strong parasite growth and stealthy invading strategy which might largely control host plant without triggering its specific defense reaction.

### Pathogenesis-related protein activity in “Zenith” roots during early infection stage

**Figure 2:**
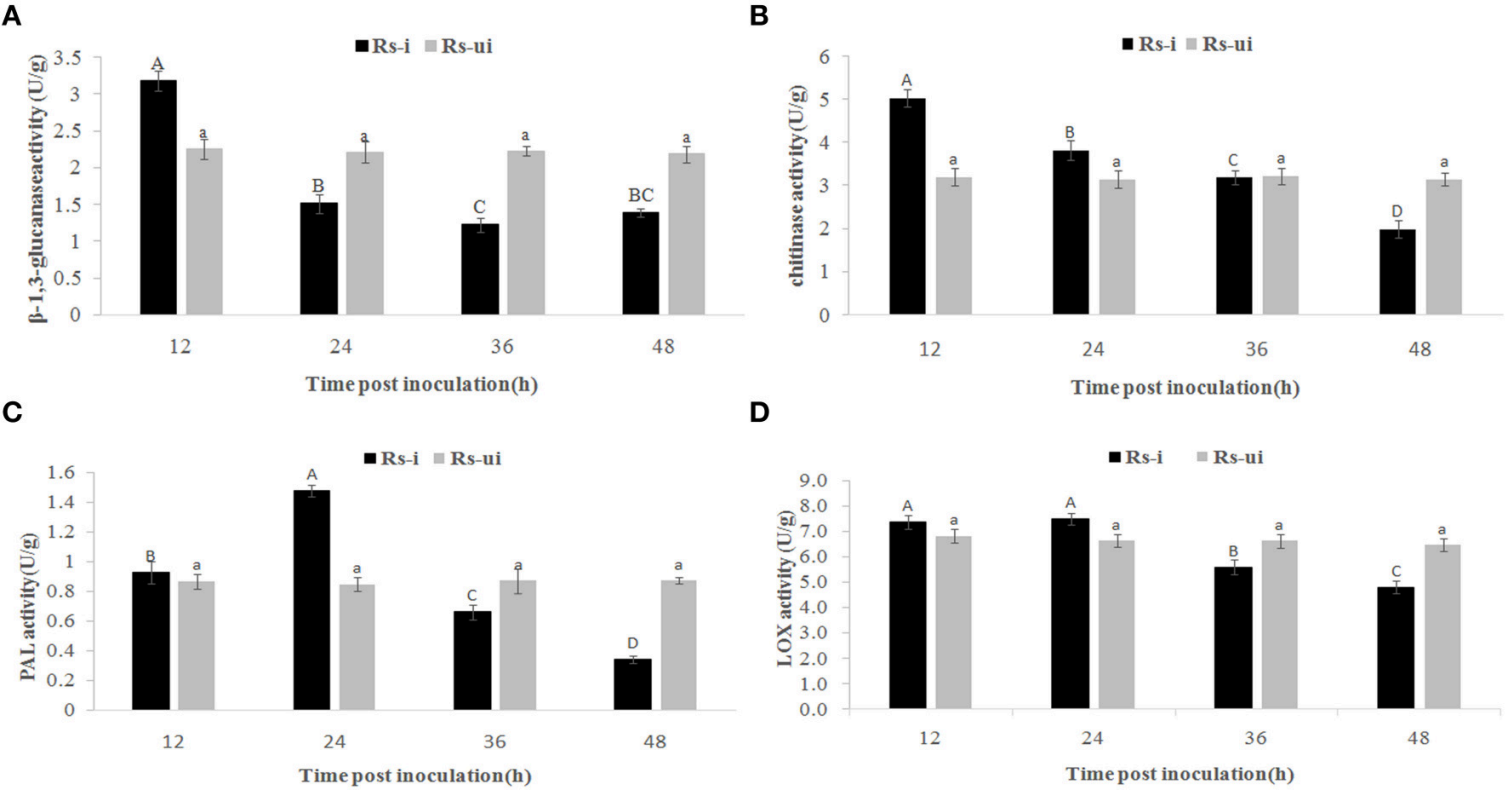
***Rs*-i: *Rhizoctonia solani* AG1 IA strain inoculated “Zenith” root, *Rs*-ui: *Rhizoctonia solani* AG1 IA strain un-inoculated “Zenith” root**. The enzyme activities with same upper case letter or lower case letter is not significantly different (*p* < 0.05). Errors bars represent ±SD (standard deviations) of three independent replications. **(A)** β-1,3-glucanase activity between *Rs*-ui and *Rs*-i “Zenith” roots; **(B)** Chitinase activity between *Rs*-ui and *Rs*-i “Zenith” roots; **(C)** Phenylalanine ammonialyase (PAL) activity between *Rs*-ui and *Rs*-i “Zenith” roots; **(D)** Lipoxygenase (LOX) activity between *Rs*-ui and *Rs*-i “Zenith” roots.

Even though there is no significant morphology difference between *Rs*-ui and *Rs*-i “Zenith” roots, we still wonder whether “Zenith” might still conserve basic defense reaction during early infection stage. Thus, we detected phenylalanine ammonialyase (PAL), β-1,3-glucanase, chitinase, and lipoxygenase (LOX) activities in *Rs*-ui and 12–48 hp*Rs*-i “Zenith” roots (Figure 2).

Compared to *Rs*-ui “Zenith” root, β-1,3-glucanase activity increased significantly at 12 hp*Rs*-i “Zenith” root while declining rapidly at 24 hp*Rs*-i “Zenith” root (Figure 2A); chitinase activity reached maximum at 12 hp*Rs*-i “Zenith” roots, while it reduced significantly at 48 hp*Rs*-i “Zenith” root (Figure 2B); PAL activity reached maximum amount at 24 hp*Rs*-i “Zenith” root while it started to reduce between 36 hp*Rs*-i and 48 hpi “Zenith” root (Figure 2C); LOX activity reached maximum amount in 12 hp*Rs*-i “Zenith” root and remained steady in 24 hp*Rs*-i “Zenith” root while it went down rapidly between 36 hp*Rs*-i and 48 hp*Rs*-i “Zenith” roots (Figure 2D).

These results mentioned above shows that a downward trend exists in all enzyme activities in *Rs*-i “Zenith” root, which indicated AG1 IA strain powerful invading ability in “Zenith” root. The substantial suppressing effect in “Zenith” root basic defense enzymes might also indicate unknown mechanism was applied by AG1 IA strain to cope with host immunity reaction.

### Transcriptome profiles of *Rs*-I “Zenith” roots and AG1 IA strain

In this transcriptome analysis, *Rs*-ui “Zenith” root was treated as T10, 12–48 hp*Rs*-i “Zenith” roots as T11, T12, T13, T14 respectively; 12–48 h infection of AG1 IA as T11′, T12′, T13′, T14′. The transcripts of AG1 IA strain were identified, after mapping with whole genome short gun data Rhisol_AG1IA (DDBJ/EMBL/GenBank under the accession code AFRT00000000). There were 96,746 unigenes for *Rs*-i “Zenith” roots and 29,066 unigenes for AG1 IA strain (Table 1).

**Table 1:** **Summary of unigenes sequenced for *Rs*-i “Zenith” roots and AG1 IA strain**.

**Length range**	***Rs*-i “Zenith” roots unigenes**	**AG1 IA strain unigenes**
200–300 (bp)	30,735 (31.77%)	2906 (10.00%)
300–500 (bp)	24,341 (25.16%)	2993 (10.30%)
500–1000 (bp)	19,001 (19.64%)	4908 (16.89%)
1000–2000 (bp)	13,978 (14.45%)	9119 (31.37%)
2000+ (bp)	8691 (8.98%)	9140 (31.45%)
Total number	96,746	29,066
Total length (bp)	76,050,816	48,597,233
N50 length (bp)	1382	2342
Mean length (bp)	786.09	1671.96

The average mapped reads ratio of *Rs*-i “Zenith” root and AG1 IA strain unigenes in is 79.74 and 91.47% respectively (Table 2), which means RNA-seq quality in this research was reliable, therefore unigenes can be used in later analysis.

**Table 2:** **Mapping information of *Rs*-I “Zenith” roots and AG1 IA strain**.

**Sample**	**Clean reads**	**Mapped reads/ratio**	**Sample**	**Clean reads**	**Mapped reads/ratio**
T10	19,541,227	15,089,687 (77.21%)			
T11	17,686,573	13,695,082 (77.43%)	T11′	2,309,941	2,106,143 (91.18%)
T12	22,097,212	17,272,601 (78.16)	T12′	1,812,085	16,59,019 (91.55%)
T13	17,253,833	13,869,193 (80.38%)	T13′	7,716,629	7,073,835 (91.67%)
T14	12,987,157	11,108,616 (85.53%)	T14′	14,307,608	13,086,997 (91.47%)

*Rs-ui “Zenith” root was treated as T10, 12–48 hpRs-i “Zenith” roots as T11, T12, T13, T14 respectively; 12–48 h infection of AG1 IA as T11′, T12′, T13′, T14′*.

The differential expressed unigenes (DEG) in *Rs*-I “Zenith” roots during early infection were acquired by comparing normalized expression level between seven pairs (For *Rs*-i “Zenith” root: T10 vs. T11, T10 vs. T12, T10 vs. T13 and T10 vs. T14; For AG1 IA strain: T11′ vs. T12′, T11′ vs. T13′, T11′ vs. T14′). For *Rs*-i “Zenith” root, there were 7059 (6196 up-regulated, 863 down-regulated), 6804 (5678 up-regulated, 1126 down-regulated), 11,000 (10,867 up-regulated, 133 down-regulated), 12,511 (12,511 up-regulated) DEGs respectively in sample T11 to T14; there were 428 (275 up-regulated, 153 down-regulated), 395 (235 up-regulated, 160 down-regulated), 553(343 up-regulated, 210 down-regulated) DEGs respectively in sample T12′–T14′.

### Validation of unigenes expression profile

To validate the reliability of transcriptome data and the *de novo* assembly results, we randomly selected six unigenes of *Rs*-i “Zenith” root for RT-qPCR using gene-specific primers to quantify the gene expression changes detected in the transcriptome analysis. The detail information of randomly selected unigenes were as follows, *methionine aminopeptidase 2A* (Group2_Unigenes_BMK.50361), *ribosomal RNA processing protein 1*(Group2_Unigenes_BMK.35545), abscisic acid 8′-hydroxylase (Group2_Unigenes_BMK.11436), *peroxidase 5* (CL10382Contig1), *light-inducible protein CPRF2* (CL5733Contig1), *cytochrome c oxidase subunit 1* (CL669Contig1). The result indicated that the expression profiles of most selected genes quantified by RT-qPCR were in agreement with those results deprived from RNA-seq (Supplementary Image 1), except *methionine aminopeptidase 2A* and *peroxidase 5* expression profiles in 48 h showing different trend compared to RNA-seq data. These differences might be caused by several factors in transcriptome process which was further elucidated by Rubio (Rubio et al., 2015). Therefore, RT-qPCR results indicated that the sequencing results were reliable.

### Functional classification of DEGs in “Zenith”

In order to find out which metabolic processes represented the most fluctuated DEGs in *Rs*-i “Zenith” roots during AG1 IA strain early infection, GO and KEGG databases were used to term the DEGs in “Zenith.”

**Figure 3:**
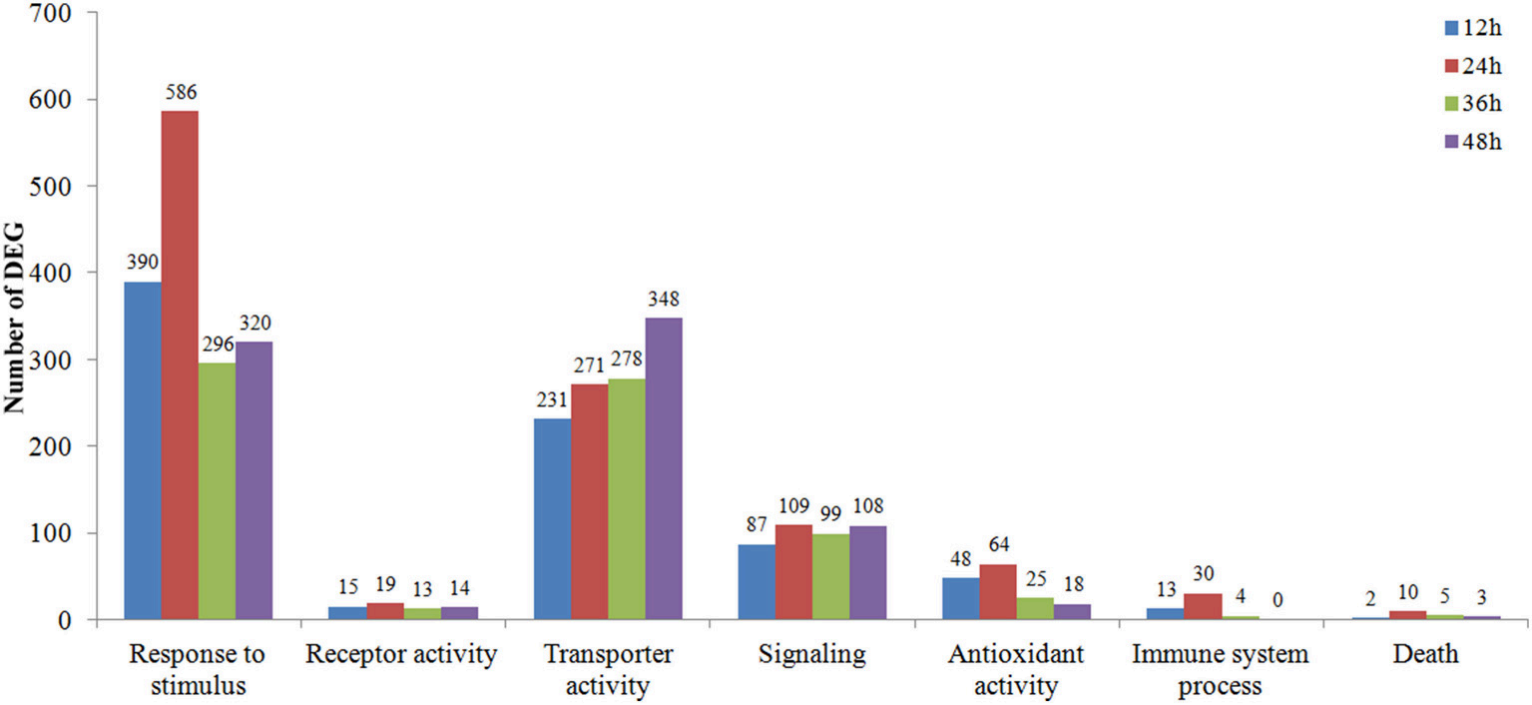
**Vertical axis represents the number of 12–48 hp*Rs*-I “Zenith” roots DEGs in contrast to those in *Rs*-ui “Zenith” root; Horizontal axis represents the metabolic pathway in GO database classification**.

The DEGs clustered into seven possible (Response to stimulus, Receptor activity, Transporter activity, Signaling, Antioxidant activity, Immune system process, Death) plant-fungal interaction related GO term assignments were compared among time course *Rs*-i “Zenith” roots (Figure 3). A general increasing to decreasing trend of DEGs number was fund in most GO term assignments except a constant rising trend in “transporter activity” and “signaling.” While comparing the number of DEGs in 24 hp*Rs*-i “Zenith” root to that in 12 hp*Rs*-i “Zenith” root, “Death” was 5 times; “Response to stimulus” was 1.5 times; “Antioxidant activity” was 1.25 times; “Signaling” was 1.25 times; and “Immune system process” was 2.3 times. These results might indicate 24 h is a critical time point for plant anti-fungal behavior activation. The number of DEGs in 36 hp*Rs*-i “Zenith” root than that of in 24 hp*Rs*-I “Zenith” root showed that “Response to stimulus” was sharply declined 50%; “Antioxidant activity” was sharply declined 61%; “Immune system process” was sharply declined 87%. Noteworthy, comparing to 36 hp*Rs*-i “Zenith” root, the number of DEGs from 48 hp*Rs*-i “Zenith” root in “Immune system process” surprisingly dropped to 0 and the steady decrease was also found in “Antioxidant activity” and “Death” processes, which possibly infers that during 36–48 h the plant is undergoing a universal repression in immune reaction to AG1 IA strain invasion.

**Figure 4:**
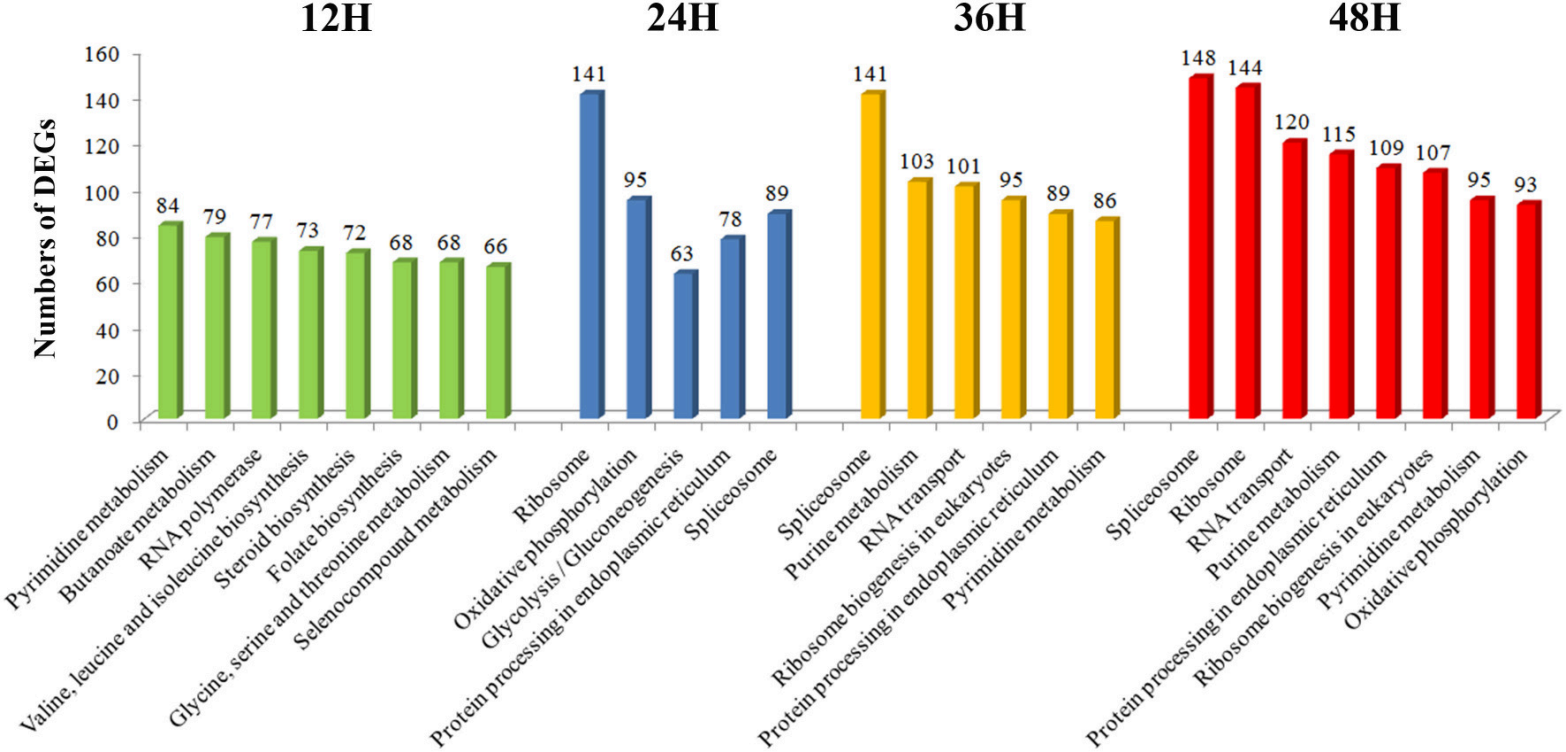
**Vertical axis represents the number of DEGs per pathway in KEGG annotation**.

During 12–48 hp*Rs*-i “Zenith” root, the number of DEGs showing most prevalent in KEGG pathways were also compared (Figure 4). The number of DEGs among the most popular KEGG pathways in 12 hp*Rs*-i “Zenith” root showed no significant difference. While in 24 hp*Rs*-i “Zenith” root, the DEGs number in “Ribosome” (protein synthesis), “Oxidative phosphorylation” (signal transduction), and “Spliceosome” (protein synthesis) pathways increased rapidly which might imply plant swift responding to AG1 IA strain infection by deploying signaling transduction and protein synthesis. Between 36 and 48 hp*Rs*-i “Zenith” roots, “Spliceosome,” and “RNA transport” (translation) pathways become the dominant in DEGs number, which indicates possible basal protection among plant pathogen related protein still works in the late stage of AG1 IA strain early invasion.

In general, according to the GO and KEGG classification the number of DEGs among 12 and 48 hp*Rs*-i “Zenith” roots dynamically changed especially in signal transduction, gene translation and protein synthesis trough extension time in AG1 IA strain invasion.

### “Zenith” resistance unigenes classification

In order to dig out more information about how “Zenith” root reacted to the early infection of AG1 IA strain, PRGdb was used to predict possible R genes in “Zenith” root.

**Figure 5:**
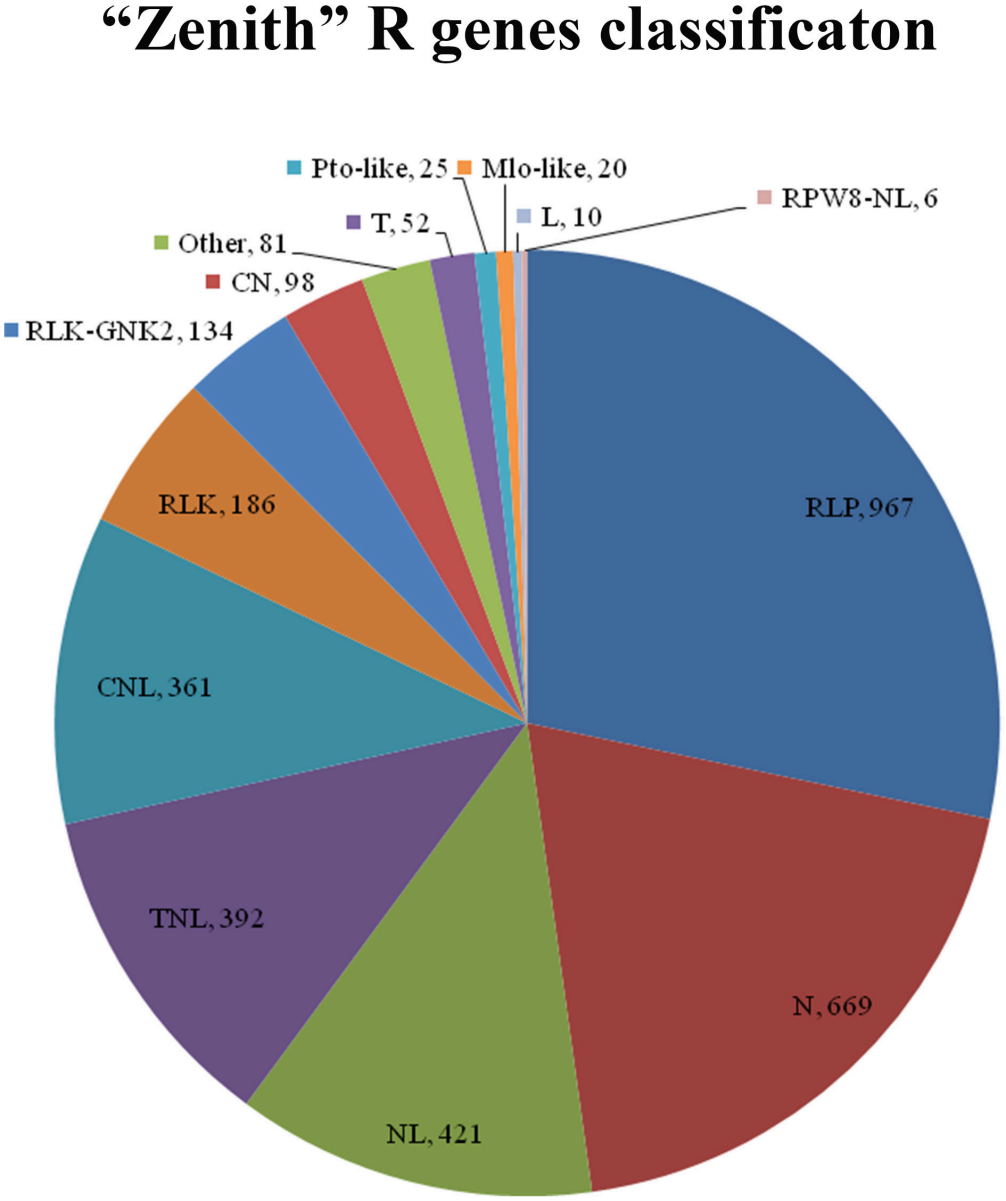
**CN, Contains coiled-coil (CC) and NBS domains; CNL, Contains a central nucleotide-binding (NB) subdomain with a leucine-rich repeat (LRR) and a CC structure as 5′ and 3′ terminal; Mlo-like, Mlo-like resistant proteins; N, Contains NBS domain only, lack of LRR; NL, Contains NBS domain at N-terminal and LRR at the C-terminal but lacks of CC domain; Pto-like, Pto-like resistant proteins; RLK, Receptor like Kinases, consisting of an extracellular leucine-rich repeat region (eLRR); RLK-GNK2, RLK class with additional domain GNK2; RLP, Receptor Like Proteins consists of a leucine-rich receptor-like repeat which is characterized as a short cytoplasmic transmembrane region without kinase domain; RPW8-NL, Contains NBS, LRR and RPW8 domains; T, Contains a Interleukin-1 Receptor (IL-1R) called TIR domain and lacks of LRR or NBS; TN, Contains TIR and NBS domains; TNL, Contains a NB subdomain with a LRR domain as C-terminal and a TIR domain as N-terminal; Other, consists of a miscellaneous set of R proteins that do not fit into any of the known classes, but that has resistance function**.

Total 3422 unigenes of “Zenith” root were predicted and classified into 14 groups which is 967, 667, 421, 392, 361, 186, 134, 98, 81, 52, 25, 20, 10, and 6 unigenes for RLP, N, NL, TNL, CNL, RLK, RLK-GNK2, CN, Other, T, Pto-like, Mlo-like, L, and RPW8-NL class respectively (Figure 5). Five classes (RLP, 967 unigenes; N, 669 unigenes; NL, 421 unigenes; TNL, 392 unigenes; and CNL, 361 unigenes) among 14 classes mentioned above possessed almost 82 percent total quantity of putative R genes in “Zenith” root (Figure 5).

The RLP class is previously recognized as a pattern recognition receptor which conducts pathogen/microbe associated molecular pattern (PAMP/MAMP) triggered immunity (PTI/ MTI) to detect a broad range of pathogens (Sekhwal et al., 2015). Among 967 RLP unigenes in “Zenith” root, 253 unigenes showed high similarity to STRUBBELIG-receptor family proteins, Leucine-rich receptor like protein kinase family proteins, Leucine-rich repeat protein kinase family proteins and Leucine-rich repeat transmembrane protein kinase family proteins. The canonical N class gene in “Zenith” root contains 27 unigenes encoding ABC-2 type transporter family proteins, 277 unigenes encoding pleiotropic drug resistance proteins. The typical NL class gene in “Zennith” root possesses 7 HOPZ-ACTIVATED RESISTANCE 1 type proteins, 23 unigenes encoding LRR and NB-ARC domains-containing disease resistance proteins, 80 unigenes encoding NB-ARC domain-containing disease resistance proteins. The TNL class gene of “Zenith” root also exhibits 12 typical unigenes encoding WRKY transcription factor proteins, which is believed to be the key component in mediating the innate immune reaction during pathogen infection (Maekawa et al., 2011).

In general, “Zenith” root possesses various kinds of R gene which are crucial for PAMP triggering immunity in other identified species; in addition, expression of these R genes might answer the enhancement of pathogenesis-related protein activity at 24 hp*Rs*-i “Zenith” root.

### AG1 IA strain unigene classification

**Figure 6:**
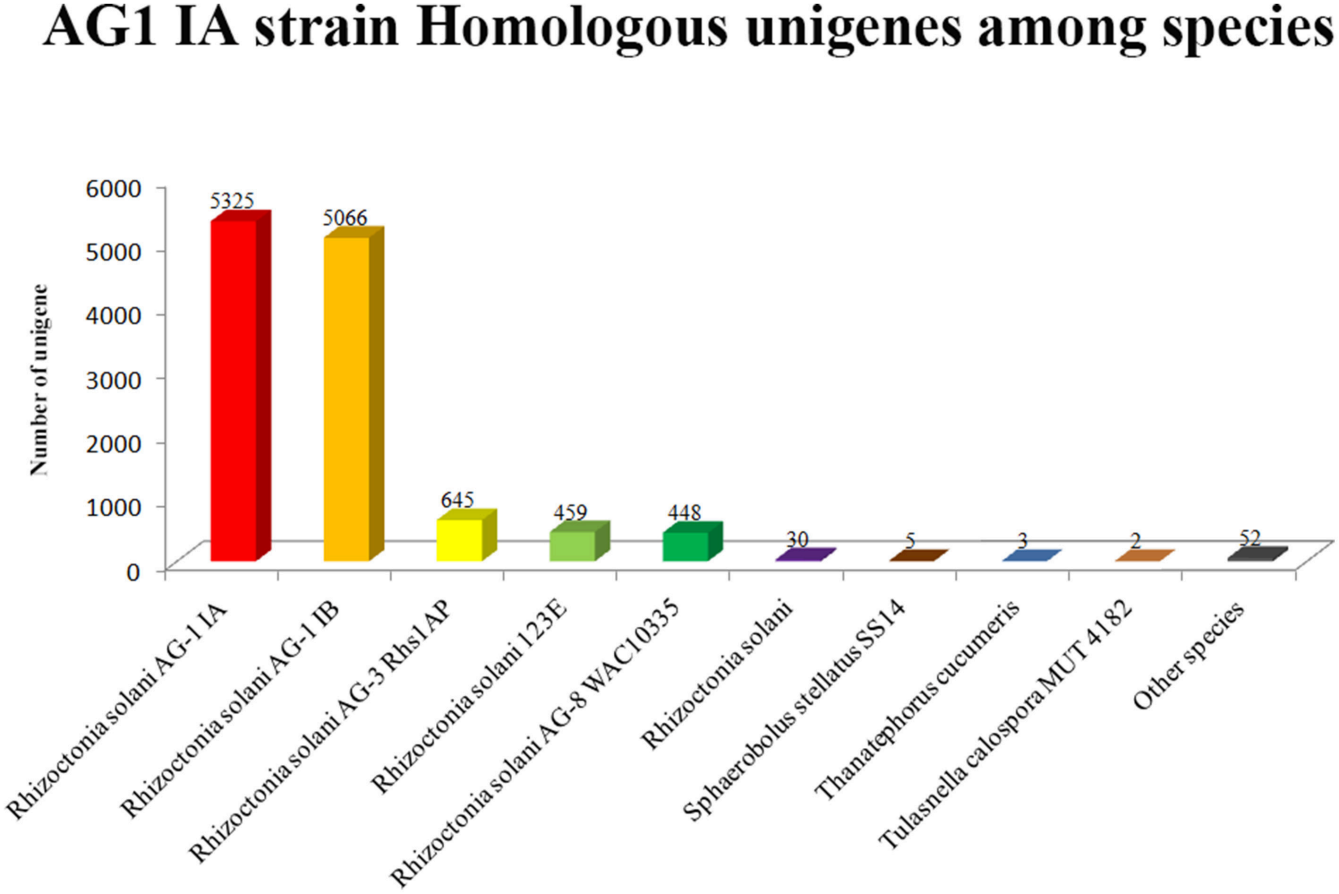
**Horizontal axis represents the name of different fungal species**.

Based on Nr database annotation, the AG1 IA strain homologous unigene were identified among different species (Figure 6), while most abundant number of homologous unigene was shared between AG1 IB strain and AG1 IA strain. Other *R. solani* species like AG3 Rhs1AP, 123E, and AG8 WAC10335 also possess large number of homologs unigene with AG1 IA strain, which might indicate close relationship in their evolution and similar parasite life style.

According to PHI database annotation, 1508 unigenes of AG1 IA strain during infection were identified and classified into different pathogen species as well as related diseases (Table 3). Total 37 pathogen species and relative diseases in different host plants were discovered. There were 386 and 224 unigenes of AG1 IA strain found respectively similar to *Fusarium graminearum* (causing fusarium ear blight in *Zea mays*) and *Magnaporthe oryzae* (causing rice blast in *Oryza sativa*) respectively, which are both abominable crop disease bringers worldwide.

**Table 3:** **General information of AG1 IA strain unigenes annotation in PHI database**.

**Pathogen species**	**Disease name**	**Number of host species**	**Number of unigene predicted in pathogenicity**
*Fusarium graminearum*	Fusarium ear blight	1	386
*Magnaporthe oryzae*	Rice blast	2	224
*Cryptococcus neoformans*	Fungal meningitis	2	160
*Ustilago maydis*	Corn smut	1	150
*Candida albicans*	Candidiasis	3	105
*Aspergillus fumigatus*	Aspergillosis	1	81
*Botrytis cinerea*	Gray mold rot	6	36
*Cochliobolus carbonum*	Leaf spot	1	21
*Fusarium verticillioides*	Maize ear rot	1	21
*Phaeosphaeria nodorum*	Glume blotch	1	20
*Leptosphaeria maculans*	Phoma stem canker	3	17
*Colletotrichum lindemuthianum*	Anthracnose	1	16
*Colletotrichum gloeosporioides*	Anthracnose	1	14
*Alternaria alternate*	Citrus brown spot	2	13
*Fusarium oxysporum*	Vascular wilt	1	12
*Colletotrichum orbiculare*	Cucumber anthracnose	1	11
*Beauveria bassiana*	White muscardine disease	1	10
*Mycosphaerella graminicola*	Septoria leaf blotch	1	9
*Colletotrichum gloeosporioides* f. sp. aeschynomenes	Anthracnose disease	1	8
*Sclerotinia sclerotiorum*	White mold/stem rot	3	8
*Cladosporium fulvum*	Leaf mold	1	7
*Colletotrichum lagenarium*	Anthracnose	1	7
*Septoria lycopersici*	Leaf spot	1	6
*Cercospora nicotianae*	Frog-eye leafspot	1	5
*Cochliobolus heterostrophus*	Southern corn leaf blight	1	5
*Nectria haematococca*	Root rot	2	5
*Pyrenochaeta lycopersici*	Corky root rot of tomato	1	5
*Erwinia amylovora*	Fire blight	1	4
*Fusarium oxysporum* f. sp. Lycopersici	Vascular wilt	1	4
*Cercospora zeae-maydis*	Maize gray leaf spot	1	3
*Penicillium digitatum*	Green mould rot	1	3
*Salmonella enterica*	Gastroenteritis	1	3
*Stagonospora nodorum*	Wheat glume blotch disease	1	3
*Claviceps purpurea*	Ergot	1	2
*Colletotrichum graminicola*	Maize anthracnose	1	2
*Gloeocercospora sorghi*	Leaf spot	1	2
*Rhynchosporium secalis*	Leaf blotch of barley	1	2

### Fungal virulence synthesis

Since virulence and its associated protein synthesis is the crucial step in strengthening the pathogenicity during pathogen-host infection, we found seven homologous unigenes matched to PHI database (*aroA, ClpV, Fgp1, FSR1, VE1, GGT, CRG1*) encoding proteins which enhanced the symptom of certain diseases in different host species.

*aroA* gene encoding 3-phosphoshikimate-1-carboxyvinyltransferase which plays a key role in production of pigment virulent via the two-component regulatory system is required for the symptom development of panicle blight of rice caused by *Burkholderia glumase* (Karki and Ham, 2014). Also putative *aroA* gene expression in AG1IA strain showed 4.4 and 3 times up-regulation at 24 and 48 h post inoculation respectively which was consistent with the disease symptom development in this case. *ClpV* gene is a member of AAA^+^ (ATPases associated with various cellular activities) protein family, which fuels the type VI secretion system by forming oligomeric complex to export the effector protein and amplify the pathogenicity (Kapitein et al., 2013). Human pathogenic fungi possessing *Fgp1* gene is orthologous to *WOR1* gene in *Fusarium oxysporum* causing devastating loss in wheat and barley which acts as the critical regulator in trichothecene toxin biosynthesis and improves the infection efficiency in breaching rachis node of spikelets (Jonkers et al., 2012). *FSR1* gene encoding protein characterizing with multiple domains (a caveolin binding domain, a coiled-coil structure, a camodulin-binding motif, and WD40 repeats) in *F. graminearum* acts directly in pathogenesis and sexual reproduction in homothallic fungus (Shim et al., 2006). *VE1* gene is one member of *velvet* genes controlling fumonisin biosynthesis, which enhances development of disease symptom in maize ear rot (Myung et al., 2012). In AG1 IA strain, this homologous unigene expression was 1.6 times down-regulated at 48 h post inoculation compared to 12 h, which might indicated the pathogenicity of AG1 IA strain might rely on multiple toxins synthesis. *GGT* gene encoding *Glutamyltranspeptidase* in *Helicobacter suis* is acted as a converter in depletion of glutathione (GSH) and glutamine (Gln) which results in apoptosis or necrosis of gastric epithelial cells *in vitro*. While, recent research declaimed that this kind of disease was cured by re-supplying the pathogenesis fungal with excessive GSH or Gln, which largely attenuated the fungal pathogenesis (De Bruyne et al., 2016). *CRG1* gene of *Cercospora nicotianae* causing leaf spot in crop acts as a critical component in synthesis of perylenequinone cercosporin toxin (Chung et al., 2003).

Taken together, during AG1 IA strain infection in “Zenith” root, AG1 IA strain harbors multiple genes which enhance the toxins biosynthesis in global major crop disease fungal species. These genes exhibit high expression or constitutive expression during infection process. Therefore, further research in discovering whether an upper regulation mechanism controlling virulence synthesis of AG1 IA strain can be found.

### Signaling and stress tolerance of AG1 IA strain during infection

Except AG1 IA strain as a necrotrophic fungus with impressive ability to produce multiple kinds of toxins in “Zenith” root infection, another prerequisite factor in its powerful infection is the perception of host environmental signal including immune reaction or phytoalexin export, which largely improves survival of the pathogen. In this study, total eight unigenes matched to PHI database (*HOG1, KPP4, RIC8, PKA1, ABC3/4, HYR1*, and *Skn7*) were found with constitutive expression in AG1 IA strain during infection, which are involved in host environmental signal perception and fungal stress tolerance.

*KPP4* gene encoding protein is the key component in Mitogen-activated protein kinase (MAPK) cascade (required for synthesis of dikaryon during *Ustilago maydis* invasion), which is activated after pheromone perception and further regulates the downstream pathogenic development gene expression (Muller et al., 2003). *Ric8* gene encodes a 480 amino acid novel protein regulating GTP-binding protein (G-protein) signaling is involved in several traits like sporulation, sexual development and plant infection of *M. oryzae* (Li et al., 2010). *PKA1* gene encoding protein is a key member of PKA catalytic module which is also the important component in comprising the cAMP/PKA pathway (regulates fungal capsule production, mating and virulence). Mutation of *pka1* gene in *cryptococcosis* resulted in reduced formation of capsule, melanin, and attenuated virulence in mouse infection model (Geddes et al., 2015). *HOG1* gene encoding protein is a major regulator of fungi in adaptive responses of host immune stress, which interacts with phosphorylated component of cell cycle transcriptional machinery to control cell morphogenesis via G_1_ cyclin expression (Gonzáález-Novo et al., 2015). *HYR1* gene encoding protein acts as a scavenger in detoxifying ROS (reactive oxygen species) generated by host plant and repressing basal immune response in host plant (Huang et al., 2011). ABC3 or ABC4 gene encoding protein is a member of well-known ATP-binding cassette (ABC) transporter protein family which is capable of couple the binding and hydrolysis of ATP to efflux a variety of compounds to keep host-derived antifungal substance within host cell (Gupta and Chattoo, 2008). ABC3 protein in *M. oryzae* improves the host penetration step during pathogenesis by functioning in plasma of appressoria (Patkar et al., 2012). *Skn7* gene encoding protein in yeast SLN1 pathway is a determinant in fungal virulence and stress regulator in fungal development via histidine kinase-based phosphorely machinery especially in oxidative stress created by host-immune reaction, which helps enhance the survival of fungal cell (Fassler and West, 2011).

All in all, AG1 IA strain controls a set of host plant basal immune response repress protein network, which subtly knocks down the defense reaction of host and help cell surviving in inhospitable environment, therefore these key factors provide necessary condition in toxin synthesis *in vivo*.

## Discussion

### “Zenith” root anti-fungal resistance

To further understand dramatically changing in immune system process of *Rs*-i “Zenith” root during AG1 IA strain invasion, potential DEGs in 12–48 hp*Rs*-i “Zenith” roots involved in anti-fungal reaction including cell wall structure biosynthesis, oxidative burst, phytoalexin in synthesis were found with Pfam annotation (Supplementary Table 1).

Total 31 DEGs encoding cytochrome P450 family enzyme which were involved in reinforced synthesis of ligin (Nelson, 2011) and three DEGs encoding cinnamoyl-CoA reductases catalyzing the key step in biosynthesis of monolignol (key component of ligin) (Tu et al., 2010) were found. Total 11 DEGs encodingagmatine-coumaroyltransferases which catalyze the last step in biosynthesis of hydroxycinnamic acid amides and enhance leaf toughness against elongation of fungal hypha (Muroi et al., 2012) were found. Thirty-five DEGs encoding glycine-rich cell wall structural proteins which increase callose level in plant vasculature and perturb the fungal metabolism transportation through plasmodesmata (Ueki and Citovsky, 2005) were discovered. Three DEGs encoding O-acyltransferases WSD1 catalyzing wax ester (key component of cuticle) which protects plant against fungal invasion (Li et al., 2008) were found both up-regulated during the whole early invasion stage. Seven DEGs encoding xylanase inhibitor proteins which suppress xylanase activity and restrain pathogen cell wall synthesis (Wu et al., 2013) were found up-regulated during early infection (12–24 h). Fifteen DEGs encoding glucanendo-1,3-beta-glucosidases which hydrolyze beta-1,3 glucan and retain fungal growth rate (Beffa et al., 1993) were dynamically changed during 24–36 hp*Rs*-I “Zenith” roots. Eight DEGs encoding Germin-like proteins also named as oxalate oxidase which degrade oxalic acid to H_2_O_2_ and retain fungal invasion (Rietz et al., 2012) were also found same asglucanendo-1,3-beta-glucosidases.

The DEGs mentioned above all showed an increasing expression ratio as the AG1 IA strain infection became severe, which indicated that the “Zenith” root preserve the perception ability during AG1 IA invasion, however these physical protections still fail to stop AG1 IA strain invasion. Thus, major specific immune reaction might be shut down or repressed by AG1 IA strain, which might infer a clue in dramatic down-regulated DEGs.

Then we screened the down-regulated DEGs in 12–48 hp*Rs*-i “Zenith” roots to discover whether any further evidence would support this opinion. Total five DEGs encoding leucine-rich repeat receptor protein kinases which are transmembrane pattern recognition receptors perceiving pathogen-associated molecular patterns and activating the expression of plant defense genes (Kemmerling et al., 2011; Roux et al., 2011) were found. Notably, three of five DEGs were down-regulated at 12 hp*Rs*-i “Zenith” roots and all five DEGs were down-regulated at 24 hp*Rs*-i “Zenith” roots and all five DEGs were totally repressed, which suggested that leucine-rich repeat receptor protein kinase might be a potential key sensor protein for “Zenith” root in AG1 IA strain invasion. Total five DEGs encoding Subtilisin-like proteases which act as plant anti-fungal signaling peptide (Pearce et al., 2010) and activates induced resistance that allows immune-related transcriptional reprogramming in host plant (Ramírez et al., 2013) were found. Interestingly, these two kinds of repressed proteases are both involved in fungal invasion perception process which might fail to activate the full immune reactions in “Zenith.” Thus, future research should pay more attention to these two kinds of enzymes including R genes mentioned above which might shed light to unveil the AG1 IA strain pathogenesis trait in “Zenith.”

### *AG1 IA* strain pathogenicity during invasion

During “Zenith” root invasion, *AG1 IA* strain self-protection mechanism against host defense reaction and disarm of host immune system should be the result of pathogenicity related genes expression. Thus, we screened potential *AG1 IA* strain unigenes participated in secondary metabolites (including carbohydrate active enzymes, transporters, toxins), secretome and candidate effectors with Pfam annotation (Supplementary Table 2).

Total 22 DEGs encoding cytochrome P450 enzymes which participate in fungal toxin or virulence synthesis (Crešnar and Petric, 2011) were found, while a down-regulated trend in 24 h post inoculation and an increasing up-regulated trend from 36 to 48 h post inoculation might indicate subtle expression control of cytochrome P450 enzymes during AG1 IA strain invasion. Total eight DEGs encoding ABC transporter family enzymes (only one ABC transporter G family member was identified) which couple hydrolysis of ATP to transport proteins including toxin, hydrolytic enzymes, and antimicrobial peptides enhancing fungal infection ability during host invasion (Lewis et al., 2012; Prasad and Goffeau, 2012; Paul et al., 2013) were discovered. To the contrary, DEGs encoding ABC transporter family enzymes showed down-regulated expression at 24–48 h post inoculation, which might indicate the unnecessary need for these enzymes in “Zenith” early infection. Total 53 DEGs encoding carbohydrate-active enzymes (CAZymes) which degrade the biosynthesis or modification of glyconjugate oligo and polysaccharide of host plant to enhance fungal infection (Charaoui-Boukerzaza and Hugouvieux-Cotte-Pattat, 2013) were found, including 46 DEGs encoding Glycosyl hydrolase (GH) family (1, 10, 12, 3, 45, 61, 7, 92, 15, 16, 25, 28, 31, 43, and 6), two DEGs encoding glycosyl transferases (GTs) and five DEGs encoding cutinases. GH family members are one main group in fungal CAZymes which decompose plant cell wall components like xylance, hemicellulose, β-1, 3-glucan or callose (polysaccharide of β-1,3-glucan) and provide basic need for fungal invasion, while the up-regulated DEGs encoding glycosyl hydrolase (GH) family in AG1 IA transcriptomes inferred multiple ways in “Zenith” cell wall degradation. Five DEGs encoding cutinases belonging to carbohydrate esterase family 5 and catalyzing cleavage of ester bonds of cutin (Roussel et al., 2014) in host plant were found in AG1 IA strain transcriptomes, which showed silent profile in rice sheath blight. Two DEGs encoding glycosyl transferases identified as effector proteins in rice sheath blight symptom (Zheng et al., 2013) were however down-regulated in 24 h but up-regulated in 48 h post inoculation, which indicated the similar trait of AG1 IA strain pathogenicity. Total six DEGs encoding fungalysin metallopeptidases which bind to chitin binding domain and degrade plant chitinases (Slavokhotova et al., 2014) were also up-regulated during AG1 IA strain invasion process. Only 1 DEGs encoding catalase which eliminates H_2_O_2_ and aid fungal survival in host living organism (Slavokhotova et al., 2014) was up-regulated during AG1 IA strain invasion. Four DEGs encoding peptidase inhibitor I9 proteins which were also identified as effector proteins in rice sheath blight symptom (Zheng et al., 2013) were both up-regulated from 24 to 48 h during AG1 IA strain invasion in this research.

According to AG1 IA strain early infection in “Zenith” transcriptomes, first major changes in AG1 IA strain genes expression lay in huge array of CAZymes to provide diversified ways for “Zenith” root cell wall degradation. While DEGs encoding cutinase found in the AG1 IA strain transcriptome inferred a possible new potential effector protein in “Zenith” infection. Similar identified effector proteins such as glycosyl transferases and peptidase inhibitor I9 proteins should also be extensively studied in the future work. Substantial number of DEGs in AG1 IA strain transcriptomes that can't be annotated should also be checked as the key candidates involved in pathogenicity in “Zenith” infection.

## Author contributions

CZ and LA contributed equally to this work; LW processed the layout of the paper and partial experiment data; PY processed partial experiment procedures; SL and CL check the spelling and processed artwork; HZ is correspondence author and funds this experiment.

### Conflict of interest statement

The authors declare that the research was conducted in the absence of any commercial or financial relationships that could be construed as a potential conflict of interest.

## Supplementary material

Supplementary Table 1 **DEGs of 12–48 hp*Rs*-i “Zenith” roots encodes anti-fungal reaction and potential effector proteins**.

Supplementary Table 2 **DEGs of *AG1 IA* strain encodes secondary metabolites (including carbohydrate active enzymes, transporters, toxins), secretome, and candidate effectors**.

Supplementary Table 3 **Primers used in validation of DEGs expression profile**.

Supplementary Image 1 **RT-qPCR validation of parts of *Z. japonica* grass root differentially expressed genes obtained with RNA-seq analysis**. FPKM (fragments per kilobase of exon per million fragments mapped) values gained with RNA-seq. Error bars represent the standard deviations for three independent experimental replicates.
