# Supplementary figures and images for: De novo Transcriptome Analysis of Rhizoctonia solani AG1 IA Strain Early Invasion in Zoysia japonica Root
Source: Front Microbiol. 2016 May 18;7:708. doi: 10.3389/fmicb.2016.00708 (PMC4870862; doi:10.3389/fmicb.2016.00708)

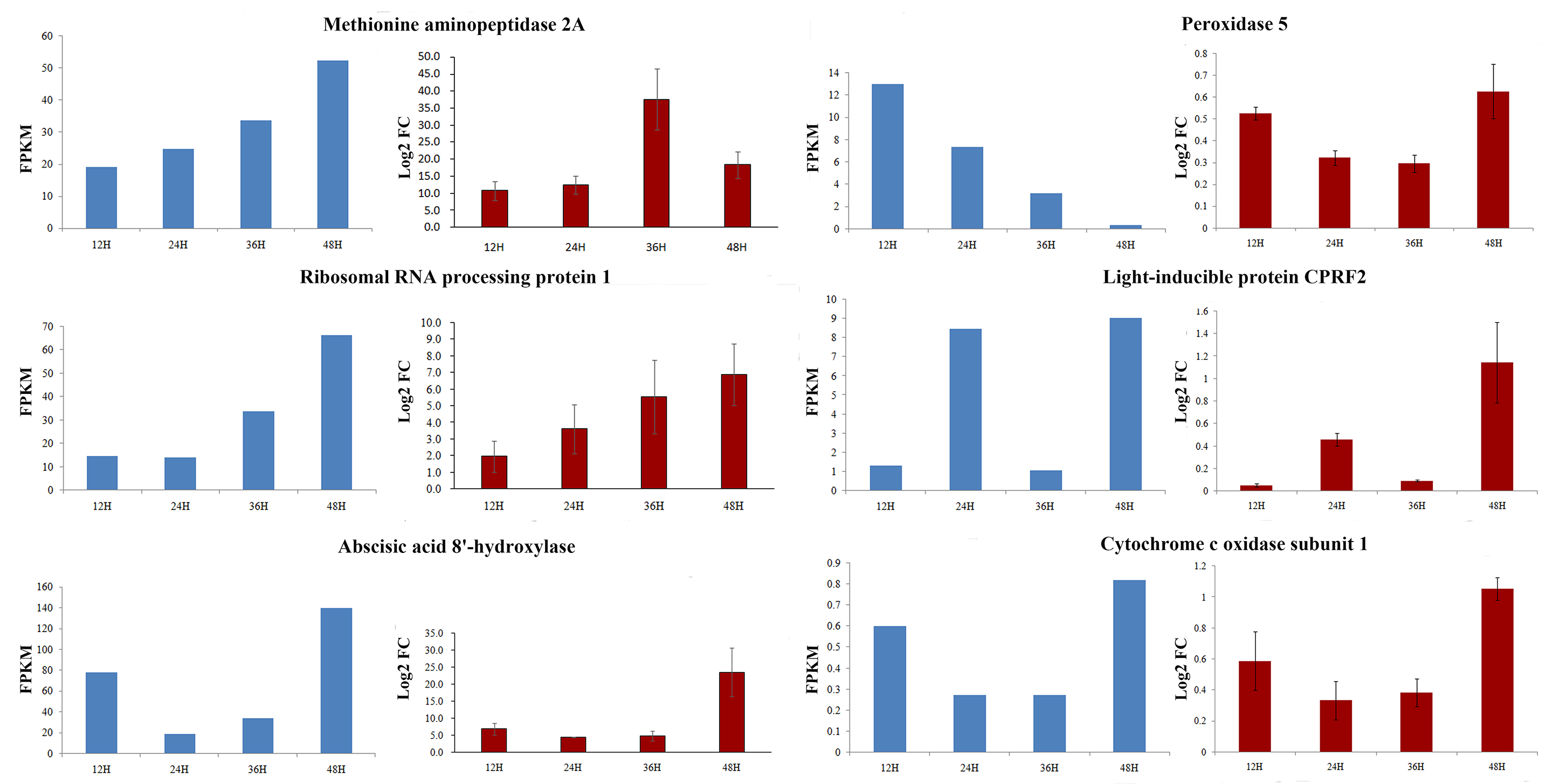

Supplement: Supplementary Image 1 — RT-qPCR validation of parts of Z. japonica grass root differentially expressed genes obtained with RNA-seq analysis. FPKM (fragments per kilobase of exon per million fragments mapped) values gained with RNA-seq. Error bars represent the standard deviations for three independent experimental replicates. [file Image1.TIF]
